# Hearing Loss and Parkinson's Disease: Insights From Human and Animal Studies

**DOI:** 10.1002/brb3.71806

**Published:** 2026-10-21

**Authors:** Xiao Yu, Wenxin Shen, Jiayu Li, Yiming Ma, Jingjing Cai, Jian Wang, Hui Wang, Shankai Yin

**Affiliations:** ^1^ Department of Otolaryngology, Shanghai Sixth People's Hospital Affiliated to Shanghai Jiao Tong University School of Medicine Shanghai China; ^2^ Otolaryngology Institute of Shanghai Jiao Tong University Shanghai China; ^3^ School of Communication Science and Disorders Dalhousie University Halifax Canada

**Keywords:** Adcyap1, auditory deprivation, hearing loss, Parkinson's disease

## Abstract

**Background:**

Hearing loss (HL) has been increasingly implicated in Parkinson's disease (PD), but the epidemiological association between them and the underlying biological mechanisms remain unclear. This study aimed to investigate the association between HL and PD and to explore potential biological links between auditory deprivation and PD‐related pathology.

**Methods:**

Data from the National Health and Nutrition Examination Survey (NHANES) and the China Health and Retirement Longitudinal Study (CHARLS) were analyzed using multivariable logistic regression, with sensitivity analyses performed in propensity score‐matched samples comprising 70 matched pairs in NHANES and 171 matched pairs in CHARLS. In parallel, an auditory‐deprivation model was established by ossicular chain interruption, while PD‐related pathology was modeled by MPTP administration once daily in three 7‐day treatment blocks (21 injections in total), with 48‐h drug‐free assessment intervals between blocks. Auditory deprivation was confirmed by auditory brainstem response. Motor function was assessed using the open field and rotarod tests, and dopaminergic neuron loss was evaluated by immunofluorescence. Striatal RNA sequencing, integrated with the GSE205450 dataset, was conducted to explore potential molecular mechanisms.

**Results:**

Self‐reported hearing impairment was associated with higher odds of PD in both NHANES (OR, 1.79; 95% CI, 1.24–2.58) and CHARLS (OR, 1.42; 95% CI, 1.01–2.01). In propensity score‐matched analyses, the association remained significant in NHANES (70 matched pairs) and CHARLS (171 matched pairs). In animal experiments, significant HL × MPTP interactions were observed for motor performance and TH‐positive dopaminergic neuron counts, with poorer outcomes in HL + MPTP mice than in MPTP‐only mice. Transcriptomic analysis identified Adcyap1 as a cross‐species candidate molecular link between auditory deprivation and PD‐related alterations.

**Conclusions:**

Self‐reported hearing impairment was associated with PD in the population‐based analyses, while experimental findings support a potential relationship between auditory deprivation and greater MPTP‐related motor and dopaminergic abnormalities.

## Graphical abstract

Population analyses showed a positive association between hearing loss and Parkinson's disease.

Experimental auditory deprivation was associated with greater MPTP‐related motor and dopaminergic abnormalities.

Adcyap1/PACAP signaling emerged as a candidate molecular link from cross‐species transcriptomic analysis.

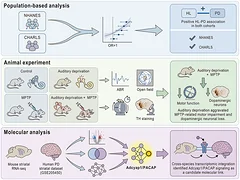

## 1 Introduction

Parkinson's disease (PD) is one of the most prevalent neurodegenerative disorders globally, secondary only to Alzheimer's disease (AD) (GBD 2016 Parkinson's Disease Collaborators 2018). World Health Organization (WHO) predicts that by 2040, neurodegenerative diseases, including PD and AD, are expected to surpass cancer as the second leading cause of death worldwide (Gammon 2014). The Global Burden of Disease (GBD) Study in 2015 identified PD as the neurological disorder with the fastest‐growing rates in the incidence and resulting disability (GBD 2015 Neurological Disorders Collaborator Group 2017). The 2021 GBD analysis further projects that by 2050, the global prevalence of PD will reach 25.2 million individuals, marking an increase of 112% compared to 2021 (Su et al. 2025). This burden is particularly relevant in China, where the age‐standardized prevalence of PD reached 245.7 per 100,000 in 2021, while an estimated 115 million people had moderate‐to‐complete hearing loss in 2015, 85.7% of whom were older than 50 years (Xu et al. 2024, Wang et al. 2025).

Although disease‐modifying treatments for PD remain limited, increasing attention has focused on the early identification of prodromal features that may facilitate timely recognition of individuals at increased risk of PD (Hu et al. 2024, Ferraz and Silva 2016, Lotankar et al. 2017, Yang et al. 2020, Chen 2010, Jr et al. 1994, Bloem et al. 2021, Daalen et al. 2022, van der Berg et al. 2024). Within this context, it is well established that PD is preceded by a prodromal period characterized by a constellation of nonspecific symptoms (Armstrong and Okun 2020). Among these, hyposmia is widely recognized as an early sensory manifestation of neurodegenerative change (Chen et al. 2021, Dintica et al. 2019), while sensory deficits more broadly have been associated with biomarkers of neurodegeneration (Paulsen et al. 2022). Importantly, sensory dysfunction in PD is not confined to olfaction: abnormalities have also been reported across peripheral, brainstem, and cortical auditory pathways (Leme et al. 2023, Jafari et al. 2020). These observations provide a rationale for investigating HL as another sensory phenotype potentially associated with PD.

In recent years, the potential association between HL and PD has garnered increasing attention. However, the results of epidemiological studies remain inconsistent. A nested case‐control study revealed that HL was significantly associated with an increased risk of developing PD (Simonet et al. 2022). A cross‐sectional study comparing subjects with and without HL indicated that HL elevated the risk of developing PD by 53% in Taiwan (Lai et al. 2014). However, two longitudinal evaluations failed to show the HL‐linked risk for PD, one with a large sample and up to 10 years follow up but self‐report of HL (Readman et al. 2023), the other with a smaller scale and shorter follow‐up but test‐documented HL (Yao et al. 2023). These inconsistent findings may partly reflect methodological heterogeneity, including differences in HL assessment, study design, follow‐up duration, sample size, and population characteristics.

In terms of potential mechanisms underlying the link between HL and PD, the evidence remains limited. Proposed pathways include neuroinflammation, altered neurotransmitter metabolism, shared genetic susceptibility, and auditory‐deprivation‐related plasticity within corticostriatal circuits (Li et al. 2021). Dopaminergic modulation of auditory responses suggests biological coupling between the auditory system and basal‐ganglia circuitry (Jafari et al. 2020), while experimental auditory deprivation has been shown to produce persistent functional alterations in the sensory striatum (Mowery et al. 2017). These observations provide a plausible framework in which reduced auditory input may be associated with altered dopaminergic vulnerability, although direct causality remains unproven. Mendelian randomization analysis has revealed overlapped genetic loci between HL and PD (Zhang et al. 2025), although no significant causal association was detected. Nevertheless, none of these proposed mechanisms has been conclusively established (Li et al. 2021). Accordingly, the epidemiological component of this study assesses self‐reported hearing impairment as captured by the cohort datasets, whereas the experimental component examines peripheral conductive auditory deprivation. These phenotypes are not measurement‐equivalent; rather, the animal model was used to provide complementary biological context regarding the consequences of reduced auditory input.

To address these epidemiological and mechanistic gaps, we investigated the relationship between HL and PD using complementary population‐based, experimental, and transcriptomic approaches. Associations between HL and PD were examined in two independent population datasets representing distinct population and ancestry backgrounds, followed by an auditory‐deprivation/MPTP mouse experiment to evaluate PD‐related motor and dopaminergic phenotypes. Striatal transcriptomic analysis was further integrated with a human PD dataset to identify candidate molecular links between auditory deprivation and PD‐related alterations.

## 2 Materials and methods

This study employed a complementary multi‐level framework integrating (1) epidemiological analyses of datasets representing distinct population and ancestry backgrounds, NHANES and CHARLS, (2) behavioral and pathological investigations in mice with auditory deprivation and/or MPTP exposure, and (3) striatal transcriptomic profiling. The two independent human datasets were used to examine the reproducibility of the HL–PD association across different populations, whereas the animal and transcriptomic analyses were used to provide experimental and molecular context for the epidemiological findings.

### 2.1 Epidemiological Population Study

In the population‐based analysis, data were obtained from two large‐scale health survey databases: the National Health and Nutrition Examination Survey (NHANES, 2005–2012 and 2015–2018) and the China Health and Retirement Longitudinal Study (CHARLS, 2011–2020). NHANES and CHARLS differ in sampling framework, survey period, questionnaire wording, and the availability and coding of several covariates. Therefore, the two datasets were analyzed separately rather than pooled or treated as directly interchangeable. Concordance between the two analyses was interpreted as cross‐database replication of the HL–PD association across distinct population backgrounds rather than as direct equivalence of the two surveys.

HL and PD were defined using cohort‐specific variables according to the data structure of each survey. PD ascertainment differed between the two datasets. In CHARLS, PD status was determined from participants’ reports of a previous physician diagnosis of PD; participants reporting such a diagnosis were classified as having PD, whereas those denying a previous diagnosis were classified as non‐PD controls. In NHANES, a direct physician‐diagnosis variable for PD was not consistently available across the survey cycles included in the analysis. Therefore, PD was operationally ascertained from self‐reported prescription‐medication information. Participants reporting use of anti‐Parkinsonian medications, including levodopa, carbidopa, pramipexole, ropinirole, amantadine, selegiline, entacapone, rasagiline, and rotigotine, were classified as having PD, consistent with previously published epidemiological approaches (Ou et al. 2026, Tu et al. 2024). Because PD ascertainment, hearing‐assessment items, survey periods, and several covariates differed between NHANES and CHARLS, the two datasets were analyzed independently rather than pooled, and concordant findings were interpreted as cross‐database replication rather than measurement equivalence.

The two population‐based databases were large‐scale and independent. To investigate the association between HL and PD, we analyzed the NHANES and CHARLS datasets separately using multivariable logistic regression models. The inclusion and exclusion criteria are shown in Figure 1. All participants with available information on both hearing status and PD status in the specified survey years were included, except those younger than 45 years or with incomplete data, mismatched ages, cochlear implant surgery, or other auditory abnormalities. Covariates included age, sex, race, education, marital status, body mass index (BMI), hearing aid use, smoking status, and comorbidities such as cancer, coronary heart disease, diabetes, lipid disorder, hypertension, and stroke, subject to dataset availability. To evaluate the robustness of the findings, propensity score matching (PSM) was further performed in each dataset as a sensitivity analysis. In both NHANES and CHARLS, PD status was specified as the dependent variable and HL status as the primary exposure variable.

**FIGURE 1:**
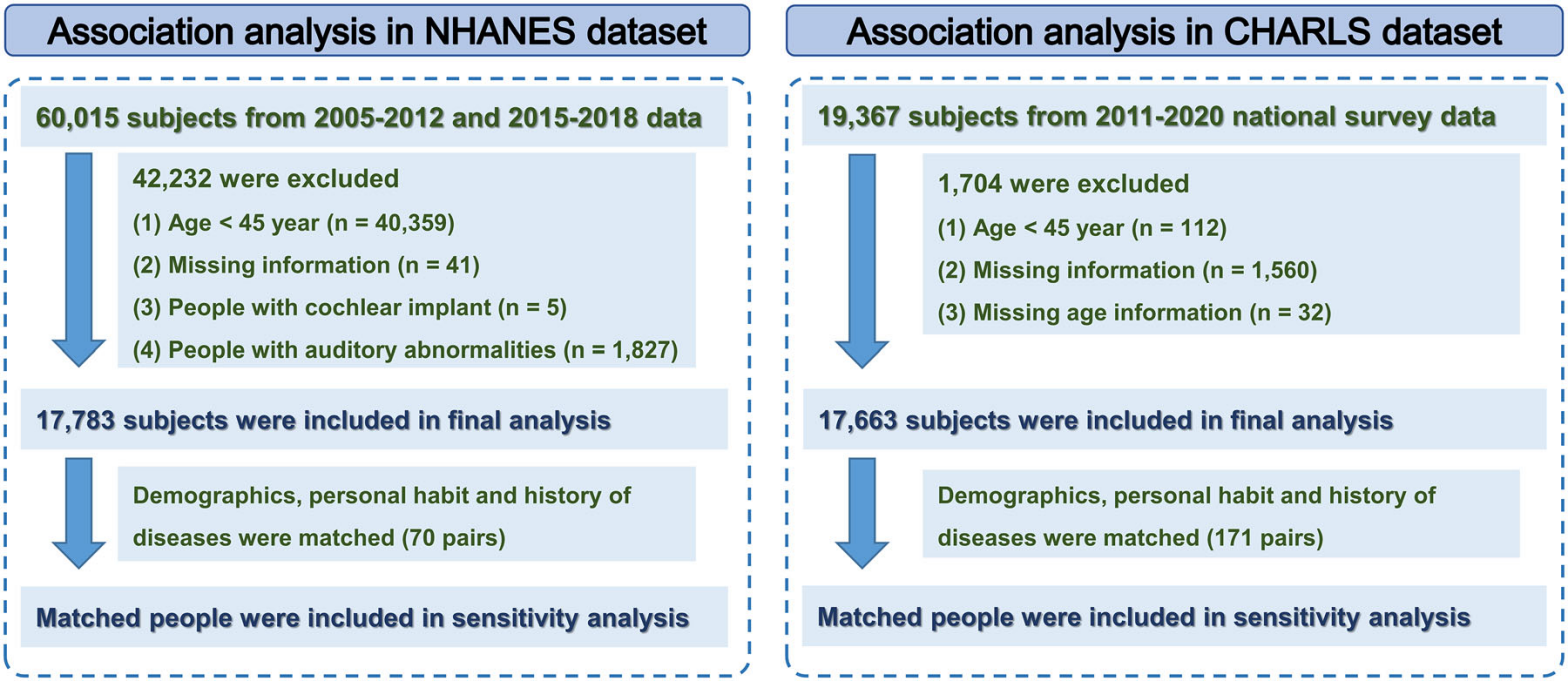
Participant inclusion and exclusion.

HL was derived from self‐reported hearing status using dataset‐specific questionnaire items. In NHANES, participants reporting a little trouble, moderate trouble, a lot of trouble, or deafness were classified as having HL, whereas those reporting excellent or good hearing were classified as not having HL. In CHARLS, participants reporting fair or poor hearing were classified as having HL, whereas those reporting excellent, very good, or good hearing were classified as not having HL. Because the underlying questions and response categories differed between the two surveys, these definitions were harmonized at the conceptual level but were not considered measurement‐equivalent. Accordingly, concordance between the two datasets was interpreted as replication of the association across different self‐reported hearing measures rather than equivalence of the hearing‐loss phenotype.

### 2.2 Animal Experiment

The design of the animal experiment was shown in Figure 2A. Briefly, 28 four‐week‐old male C57BL/6J mice were obtained from the Laboratory Animal Center of Shanghai Jiao Tong University. Before any experimental intervention, the 28 mice were randomly allocated to four groups (*n* = 7/group): (i) Control, (ii) HL, (iii) MPTP, and (iv) HL + MPTP. Auditory deprivation was induced by bilateral ossicular chain interruption, producing a conductive reduction in auditory input. This manipulation therefore represents a peripheral conductive auditory‐deprivation model rather than a model of age‐related sensorineural HL or central auditory processing disorder. 1‐Methyl‐4‐phenyl‐1,2,3,6‐tetrahydropyridine (MPTP) was used to induce PD‐related pathology. The dose, route, and 7‐day treatment block were selected on the basis of established subacute MPTP paradigms in C57BL/6 mice (Zhao et al. 2019). Specifically, MPTP was administered intraperitoneally at 25 mg/kg once daily for 7 consecutive days per treatment block. In the present study, this 7‐day regimen was incorporated into a repeated treatment–assessment protocol. Each treatment block was followed by a 48‐h drug‐free interval, after which motor performance was assessed using the open‐field and rotarod tests. When interim behavioral assessment indicated that the PD‐like motor phenotype had not yet been adequately established, the same treatment–assessment cycle was repeated. In the present experiment, mice ultimately received three 7‐day treatment blocks, corresponding to 21 MPTP injections in total, with a 48‐h drug‐free assessment interval after each block. The decision to proceed to an additional treatment block was made at the experimental‐cohort level rather than for individual animals; all mice assigned to the MPTP condition received the same number of treatment blocks. All animals were housed under standard conditions (22 ± 1°C, 12‐h light/dark cycle, lights on at 7:00 a.m.) with free access to food and water. Hearing status was examined by auditory brainstem responses (ABR). Motor ability was tested by the open field test and rotarod test. PD‐related brain pathologies were examined by immunofluorescence against specific markers for PD and by exploring specific gene expressions in RNA sequencing. All those measures were compared across the 4 groups. The prespecified exclusion criteria were death or a humane endpoint before completion of the protocol, severe procedure‐related complications preventing completion of the experiment, or technical failure precluding acquisition of a prespecified endpoint. All seven mice in each group underwent the behavioral assessments. Following completion of the final behavioral assessment, the animals in each group were assigned to one of two terminal analyses: three mice were used for striatal RNA sequencing and the remaining four mice were used for histological and immunofluorescence analyses. No animals were excluded during this allocation. Individual animal allocation and final behavioral measurements are provided in Table S7.

**FIGURE 2:**
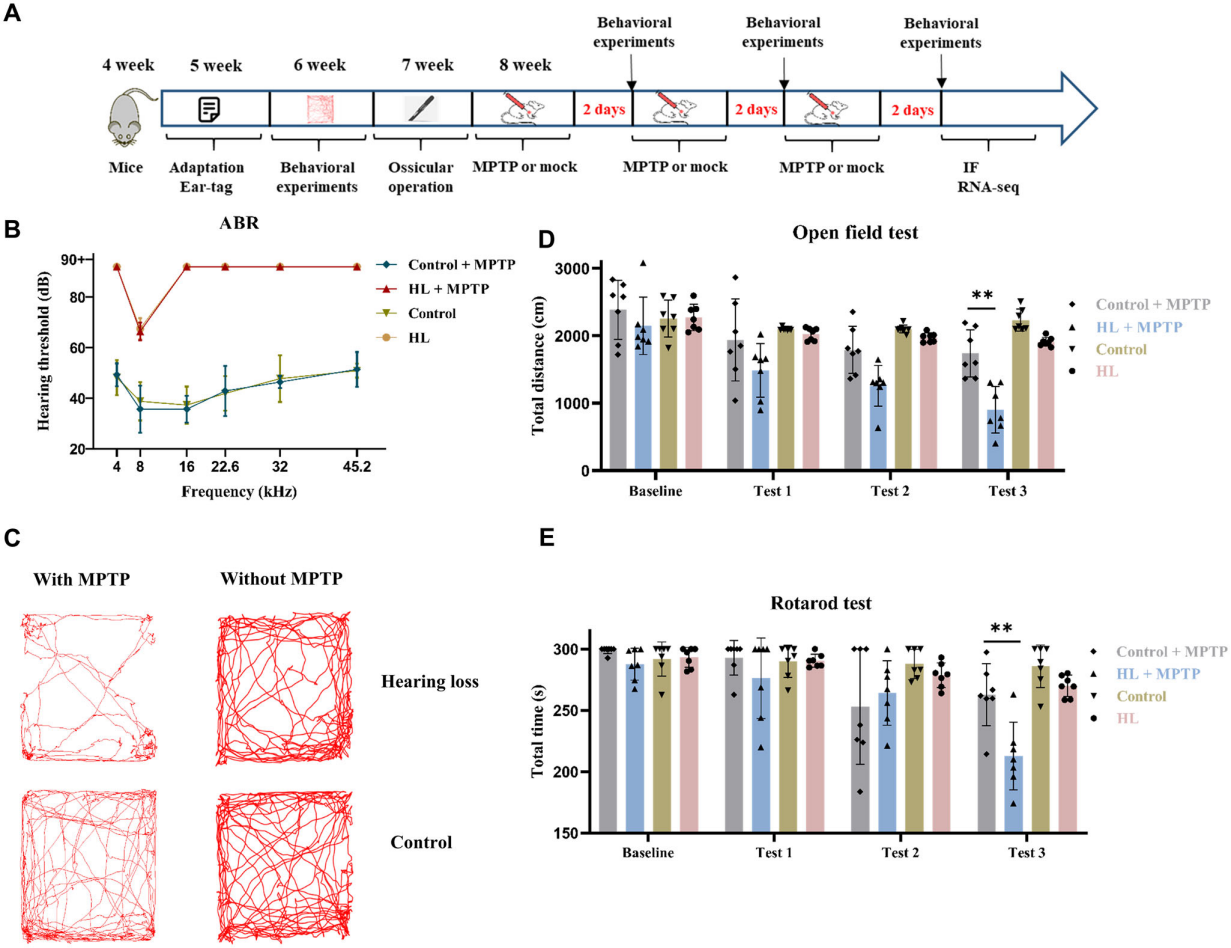
Auditory deprivation is associated with greater MPTP‐related motor impairment. (A) experimental timeline. Mice were acclimated and underwent baseline behavioral assessment before ossicular chain interruption. MPTP (25 mg/kg, i.p.) or saline was administered once daily for 7 consecutive days per treatment block. Each block was followed by a 48‐h drug‐free interval and behavioral assessment, and the treatment–assessment cycle was repeated three times in the present experiment (21 MPTP injections in total). (B) Auditory brainstem response (ABR) thresholds after ossicular chain interruption. (C) Representative open‐field trajectories. (D) Open‐field total distance across baseline and post‐treatment assessments. (E) Rotarod latency across baseline and post‐treatment assessments. Data are presented as mean ± SD, with individual data points shown (*n* = 7 mice/group). Statistical analysis was performed using two‐way ANOVA with hearing status and MPTP treatment as factors, followed by Šidák's multiple‐comparisons test. Adjusted post hoc comparisons between the MPTP and auditory‐deprivation + MPTP groups are indicated in the figure. **p < 0.001.

All experimental procedures were performed in accordance with the National Institutes of Health Guidelines on the Use of Laboratory Animals and were approved by the Laboratory Animal Center of Shanghai Jiao Tong University (A2024333‐001).

#### 2.2.1 Auditory Brainstem Response (ABR)

ABR was recorded using a TDT System 3 workstation (Tucker‐Davis Technologies, Alachua, FL, USA). C57BL/6J mice aged 6–8 weeks were anesthetized with 1% sodium pentobarbital (75 mg/kg, intraperitoneally) and placed on a thermostatically controlled heating pad maintained at 37°C within a sound‐attenuated chamber. Subdermal needle electrodes were positioned at the vertex (active), beneath the test ear (reference), and on the hind limb (ground). Acoustic stimuli were generated and controlled using SigGenRZ software (Tucker‐Davis Technologies). Tone bursts at 4, 8, 16, 22.6, 32, and 45.2 kHz were presented at a maximum level of 90 dB sound pressure level (SPL), and stimulus intensity was subsequently reduced in 5 dB SPL increments down to 0 dB SPL. The ABR threshold was defined as the lowest intensity at which a reproducible wave III could be clearly identified across the 4–45 kHz frequency range. All ABR recordings and threshold determinations were performed by an audiologist experienced in animal auditory electrophysiology. ABR was used solely to verify the auditory deprivation induced by ossicular chain interruption in mice and was not used to validate or calibrate the self‐reported hearing phenotype in the human cohorts.

#### 2.2.2 Behavioral Experiments

In this study, the open field test and rotarod test were conducted for behavioral experiments. For the open field test, mice were acclimated to the testing room for 15 min prior to the experiment. Subsequently, each mouse was placed in the center of the open field arena and allowed to explore freely for 10 min. The locomotor activity was tracked using a video‐tracking system, and the total distance traveled by the mouse was calculated. For the rotarod test, mice underwent a 3‐day adaptation and training period prior to the formal experiment. In the formal experiment, mice were placed on a rotating rod with an initial speed of 4 rpm, which was then gradually accelerated to 40 rpm over a period of 5 min. The latency to fall from the rod was recorded for each mouse. In each experiment, mice were tested 3 times, and the average latency to fall was recorded. Behavioral assessments were performed and analyzed by an investigator who was blinded to group allocation.

#### 2.2.3 Immunofluorescence Analysis

Following completion of the behavioral assessments, four mice from each experimental group (*n* = 4/group) were used for histological and immunofluorescence analyses. Brain tissues were fixed in 4% paraformaldehyde at 4°C for 24 h, dehydrated through a graded ethanol series, cleared in xylene, and embedded in paraffin. Coronal sections of the substantia nigra were cut at a thickness of 4 µm using a rotary microtome (Leica, Germany). For staining, sections were deparaffinized in xylene, rehydrated through descending ethanol concentrations, and subjected to antigen retrieval in citrate buffer (10 mM, pH 6.0) at 95°C for 20 min. After cooling to room temperature, nonspecific binding was blocked with 5% normal goat serum for 1 h. Tyrosine hydroxylase (TH), the rate‐limiting enzyme in dopamine synthesis, is widely recognized as a specific marker for dopaminergic neurons in the substantia nigra (Troshev et al. 2023). Sections were then incubated overnight at 4°C with primary antibody against TH (TH; 1:500, Abcam, UK), followed by incubation with Alexa Fluor–conjugated secondary antibodies (1:1000, Invitrogen, USA) for 1 h at room temperature in the dark. Nuclei were counterstained with DAPI (1 µg/mL, Sigma‐Aldrich). Images were captured using a fluorescence microscope (Nikon, Japan), and TH‐positive neurons in the substantia nigra were quantified using ImageJ software (NIH, USA). Before image acquisition and quantification, tissue sections were coded so that the investigator performing TH‐positive neuron quantification was blinded to experimental group allocation. Group identities were revealed only after quantification had been completed.

#### 2.2.4 RNA Sequencing

Following completion of the behavioral assessments, three mice from each experimental group were used for striatal RNA sequencing (*n* = 3 biological replicates/group). Each biological replicate consisted of dorsal striatal tissue obtained from one individual mouse, and samples from different animals were not pooled. After euthanasia, the dorsal striatum was immediately dissected and collected for RNA sequencing (Chen et al. 2019). Total RNA was extracted using TRIzol reagent (Invitrogen, USA) following the manufacturer's instructions. RNA integrity and concentration were assessed using an Agilent 2100 Bioanalyzer (Agilent Technologies, USA). High‐quality RNA samples were used to construct sequencing libraries with the TruSeq RNA Sample Preparation Kit (Illumina, USA). Libraries were sequenced on an Illumina NovaSeq 6000 platform to generate 150 bp paired‐end reads. Raw data were processed to remove adapters and low‐quality reads, and clean reads were aligned to the mouse reference genome (GRCm39) using HISAT2. Gene expression abundance was additionally calculated as fragments per kilobase of transcript per million mapped reads (FPKM) for expression visualization. Differential‐expression testing was performed on raw gene‐level count data using DESeq2 (version 1.22.2). DESeq2 internally normalized the count data and modeled gene‐wise counts using a negative‐binomial framework. Differential‐expression analyses were performed separately for the prespecified comparisons of HL versus Control and HL + MPTP versus MPTP. For each comparison, experimental group was included as the design factor, corresponding to the formula ∼ group. No batch covariate was included because all RNA samples were processed, libraries were constructed, and sequencing was performed within the same batch. Genes with |log2 fold change| > 1 and *p* < 0.05 were considered differentially expressed. FPKM values were used for expression visualization and were not used as input for DESeq2 differential‐expression testing.

For cross‐species comparison, human postmortem striatal RNA‐sequencing data and associated clinical information were obtained from the Gene Expression Omnibus dataset GSE205450, which included caudate and putamen samples from 40 control donors and 35 donors with autopsy‐confirmed PD and was generated on the Illumina NovaSeq 6000 platform (GPL24676) (Irmady et al. 2023). Genes were filtered using filterByExpr in edgeR, and differential‐expression analysis was performed using limma with voomWithQualityWeights to account for the mean–variance relationship and sample‐level differences in RNA quality. Samples were grouped according to brain region and disease status. The differential‐expression model used a no‐intercept design with age at death included as a continuous covariate, corresponding to ∼ 0 + group + death_age. PD‐versus‐control contrasts were evaluated separately for the caudate and putamen using lmFit, followed by empirical‐Bayes moderated statistics with eBayes. Multiple‐testing correction was performed using the Benjamini–Hochberg method. In this PD‐versus‐control brain differential‐expression model, age at death was included as the covariate; sex and postmortem interval were not included as additional covariates.

### 2.3 Statistical Analysis

Quantitative animal data are presented as mean ± standard deviation (SD). Behavioral outcomes, ABR thresholds, and TH‐positive neuron counts were analyzed using two‐way ANOVA, with hearing status (HL− vs. HL+) and MPTP treatment (MPTP− vs. MPTP+) as factors. The HL × MPTP interaction was evaluated, followed by Šidák's multiple‐comparisons test for comparisons between animals with and without HL within each MPTP condition. Behavioral outcomes were analyzed separately at each assessment, and ABR thresholds were analyzed separately at each tested frequency. Categorical variables were analyzed using the chi‐square test. To evaluate the independent association between HL and PD, multivariable logistic regression models were separately constructed for the NHANES and CHARLS datasets. For NHANES, full‐sample descriptive and regression analyses incorporated the appropriate survey sampling weights, strata, and primary sampling units to account for the complex multistage sampling design. Covariates included demographic, socioeconomic, and clinical factors. Odds ratios (ORs) and 95% confidence intervals (CIs) were calculated to estimate the association strength. Due to data availability, BMI and race were not included as covariates in CHARLS, and hearing aid use was not adjusted for in NHANES. Covariates were specified a priori based on clinical relevance and availability within each dataset rather than selected using data‐driven model‐selection procedures. PSM was performed as a sensitivity analysis to improve comparability between participants with and without PD. Propensity scores, representing the probability of having PD conditional on baseline covariates, were estimated separately for the NHANES and CHARLS datasets using logistic regression. In NHANES, the propensity‐score model included age group (≤ 55 vs. > 55 years), sex, race, marital status, education, smoking status, BMI, diabetes, coronary heart disease, hypertension, stroke, hyperlipidemia, and cancer. In CHARLS, the model included age group (≤ 60 vs. > 60 years), sex, marital status, education, smoking status, diabetes, coronary heart disease, hypertension, stroke, lipid disorder, and cancer. Participants with PD were treated as the focal group and were matched 1:1 to participants without PD without replacement. A caliper width of 0.20 standard deviations of the logit‐transformed propensity score was applied. Within the set of eligible control participants satisfying the propensity‐score caliper, Mahalanobis distance based on the baseline covariates was used to identify the most comparable control. Continuous age and BMI were additionally incorporated into the Mahalanobis‐distance calculation in NHANES, whereas continuous age was incorporated in CHARLS. Hearing‐loss status was not included in propensity‐score estimation or in the matching distance and therefore did not influence selection of the matched controls. Hearing‐aid use was likewise not included in the propensity‐score model because it is closely related to hearing status. Covariate balance before and after PSM was assessed using standardized mean differences (SMDs). For continuous variables, SMDs were calculated as the difference in group means divided by the pooled standard deviation, whereas for categorical variables, SMDs were based on differences in category proportions. For categorical variables with more than two levels, the maximum absolute SMD across category levels was used to summarize balance for that covariate. An absolute SMD < 0.10 was considered indicative of acceptable covariate balance. Complete pre‐ and post‐matching SMDs are provided in Table S6. Associations in the propensity score‐matched samples were estimated using conditional logistic regression to account for the matched‐pair structure. The protein–protein interaction network was constructed using the GeneMANIA database (https://genemania.org/), and gene ontology (GO) and Kyoto Encyclopedia of Genes and Genomes (KEGG) enrichment analyses were performed using the clusterProfiler package (version 4.4.4) in R. All plots and statistical analyses were conducted using GraphPad Prism and R software (version 4.2.1), and significance was defined as *p* < 0.05. Complete two‐way ANOVA and Šidák‐adjusted multiple comparisons were conducted for behavioral, ABR, and TH‐positive neuron data.

## 3 Results

### 3.1 The Epidemiological Study in Human Cohorts

#### 3.1.1 Prevalence of Self‐Reported Hearing Impairment in Participants With and Without PD

The baseline characteristics of participants in the NHANES and CHARLS datasets are summarized in Tables S1–S4 (Supplementary Material). The NHANES dataset included 17,783 participants (9038 females and 8745 males); the CHARLS dataset contained 17,663 participants (9375 females and 8288 males). We first compared the prevalence of self‐reported hearing impairment between participants with and without PD. In NHANES, the survey‐weighted prevalence of self‐reported hearing impairment was higher among participants with PD (139 of 266, 47.43%) than among those without PD (5742 of 17,517, 31.52%; *p* < 0.001). Similarly, in CHARLS, the prevalence of self‐reported hearing impairment was higher among participants with PD (74 of 181, 40.88%) than among those without PD (3577 of 17,482, 20.46%; *p* < 0.001).

#### 3.1.2 Association Between Self‐Reported Hearing Impairment and PD

To further evaluate the association between self‐reported hearing impairment and PD, multivariable logistic regression analyses were performed in the full NHANES and CHARLS samples (Table 1). In NHANES, self‐reported hearing impairment was associated with higher odds of PD after adjustment for potential confounders (OR = 1.79, 95% CI: 1.24–2.58). A similar association was observed in CHARLS (OR = 1.42, 95% CI: 1.01–2.01). PSM was subsequently performed as a sensitivity analysis, with participants with PD treated as the focal group in both datasets. Matching yielded 70 pairs in NHANES and 171 pairs in CHARLS, with all evaluated covariates achieving acceptable post‐matching balance (absolute SMD < 0.10; Table S6). In the matched NHANES sample, self‐reported hearing impairment remained associated with higher odds of PD (OR = 2.67, 95% CI: 1.04–6.81). A similar association was observed in the matched CHARLS sample (OR = 1.64, 95% CI: 1.05–2.58). These sensitivity analyses were consistent with the associations observed in the full‐sample analyses.

**TABLE 1:** Associations between self‐reported hearing impairment and Parkinson's disease in NHANES and CHARLS.

Databases	Model	OR (95% CI)	*p* value
NHANES	Full‐sample adjusted	1.79 (1.24,2.58)	0.002
	PSM‐matched	2.67 (1.04–6.81)	0.040
CHARLS	Full‐sample adjusted	1.42 (1.01, 2.01)	0.045
	PSM‐matched	1.64 (1.05, 2.58)	0.031

*Note*: Odds ratios (ORs) and 95% confidence intervals (CIs) in the full‐sample adjusted analyses were estimated using multivariable logistic regression. In the propensity score‐matched analyses, ORs and 95% CIs were estimated using conditional logistic regression to account for the matched‐pair structure. Full‐sample models were adjusted for age, sex, education, marital status, smoking status, and comorbidities; race and body mass index (BMI) were additionally adjusted for in NHANES but were not available in CHARLS, and hearing‐aid use was adjusted for in CHARLS but not NHANES. PSM, propensity score matching.

### 3.2 Animal Experiment

#### 3.2.1 Auditory Deprivation and Locomotor Deficits

To investigate the relationship between auditory deprivation and MPTP‐related motor dysfunction, mice were assigned to four experimental groups: Control, HL, MPTP, and HL + MPTP (Figure 2A). Baseline ABR thresholds were broadly comparable across the four experimental groups. No significant HL × MPTP interaction or Šidák‐adjusted within‐MPTP‐condition pairwise difference was observed at any tested baseline frequency, although an isolated main effect of the hearing‐status factor was detected at 45.2 kHz (*p* = 0.034; Figure S1 and Table S5B). Behavioral assessments at Tests 1–3 were performed 48 h after the corresponding 7‐day MPTP or saline treatment blocks. Within the animal experiment, ABR measurements confirmed the auditory deprivation induced by ossicular chain interruption (Figure 2B). Two‐way ANOVA showed a significant main effect of hearing status on ABR thresholds across all tested frequencies after ossicular chain interruption, whereas no significant HL × MPTP interaction was detected, indicating that the threshold elevation was associated with the hearing manipulation rather than MPTP treatment (Figure 2B; Table S5B).

At baseline, no significant HL × MPTP interaction was observed for either open‐field distance (F(1,24) = 0.93, *p* = 0.344) or rotarod latency (F(1,24) = 2.59, *p* = 0.121). In the open‐field test, the HL × MPTP interaction became significant at Test 2 (F(1,24) = 5.23, *p* = 0.031) and remained significant at Test 3 (F(1,24) = 6.84, *p* = 0.0152). At Test 3, HL+MPTP mice traveled a shorter distance than MPTP‐only mice (903.4 ± 345.5 vs. 1739.4 ± 349.2 cm; Šidák‐adjusted *p* < 0.0001), whereas the difference between HL‐only and control mice was not significant (*p* = 0.060).

A significant HL × MPTP interaction was also observed for rotarod performance at Test 3 (F(1,24) = 4.54, *p* = 0.0436). HL + MPTP mice showed a shorter latency to fall than MPTP‐only mice (212.7 ± 27.5 vs. 262.7 ± 25.4 s; Šidák‐adjusted *p* < 0.001), whereas no significant difference was observed between HL‐only and control mice (269.9 ± 8.7 vs. 285.9 ± 17.4 s; *p* = 0.307). These results demonstrate a significant interaction between auditory deprivation and MPTP treatment in the later‐stage motor phenotypes.

#### 3.2.2 Auditory Deprivation and TH‐Positive Cell Counts in MPTP‐Treated Mice

Immunofluorescence staining was performed to quantify TH‐positive dopaminergic neurons in the substantia nigra across the four experimental groups (Figure 3A). Two‐way ANOVA showed significant main effects of hearing status (F(1,12) = 132.21, *p* < 0.000001) and MPTP treatment (F(1,12) = 883.53, *p* < 0.000001), together with a significant HL × MPTP interaction (F(1,12) = 88.71, *p* < 0.000001). TH‐positive neuron counts were significantly lower in HL + MPTP mice than in MPTP‐only mice (15.3 ± 5.0 vs. 58.0 ± 5.1; Šidák‐adjusted *p* < 0.0001). In contrast, TH‐positive neuron counts did not differ significantly between HL‐only mice and controls (95.3 ± 2.5 vs. 99.5 ± 3.1; Šidák‐adjusted *p* = 0.306). These findings demonstrate a significant interaction between auditory deprivation and MPTP treatment for TH‐positive cell counts.

**FIGURE 3:**
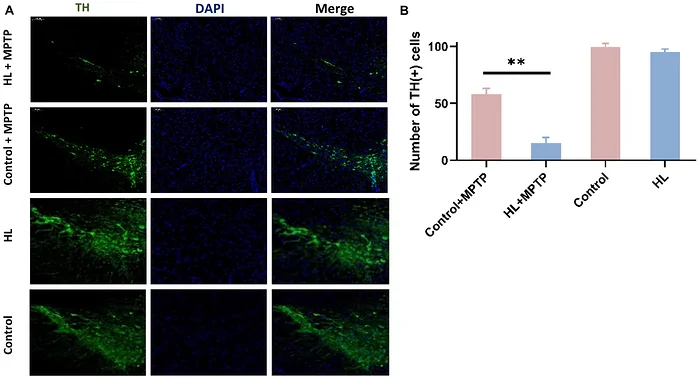
Auditory deprivation is associated with a greater reduction in TH‐positive cell counts after MPTP exposure. (A) Representative immunofluorescence images of tyrosine hydroxylase (TH, green) and DAPI (blue) in the substantia nigra from the four experimental groups. (B) Quantification of TH‐positive neurons. Data are presented as mean ± SD (*n* = 4 mice/group). Statistical analysis was performed using two‐way ANOVA with hearing status and MPTP treatment as factors, followed by Šidák's multiple‐comparisons test. Adjusted post hoc comparisons are indicated in the figure. **p < 0.001; ns, not significant.

#### 3.2.3 Transcriptomic Analysis and Candidate Molecular Links

RNA‐seq analysis of the mouse striatum showed that, in mice without MPTP exposure, HL alone induced 318 DEGs, including 42 upregulated and 276 downregulated transcripts (Figure 4A). In all MPTP‐treated mice, comparison between those with and without HL showed that HL led to 58 DEGs, 21 upregulated and 37 downregulated (Figure 4B). By intersecting these two gene sets, we identified six candidate genes whose expression was consistently altered by auditory deprivation under both non‐MPTP and MPTP conditions: five were consistently downregulated, whereas one was consistently upregulated (Figures 4C–D, Table 2). These genes represent candidate transcriptional changes associated with auditory deprivation that were observed both in the absence and presence of MPTP exposure. We subsequently reasoned that, among these six candidate genes, those also dysregulated in human PD might constitute particularly relevant candidates potentially associated with the relationship between auditory deprivation and PD‐related pathology. Therefore, we prioritized these six genes for further validation in human PD transcriptomic data. To assess the cross‐species relevance of the six candidate genes identified above, we examined their expression in human postmortem caudate and putamen samples from GSE205450 (Figures 4E–F). Five of the six candidate genes were consistently downregulated, whereas Gm6710 was consistently upregulated across the two mouse comparisons. To assess directionally concordant cross‐species overlap, the five consistently downregulated mouse candidates were compared with downregulated DEGs in human caudate and putamen samples from GSE205450. No overlapping candidate was identified in the caudate, whereas Adcyap1 was the only overlapping gene in the putamen (Figures 4G–H), thereby prioritizing Adcyap1 for further functional investigation.

**FIGURE 4:**
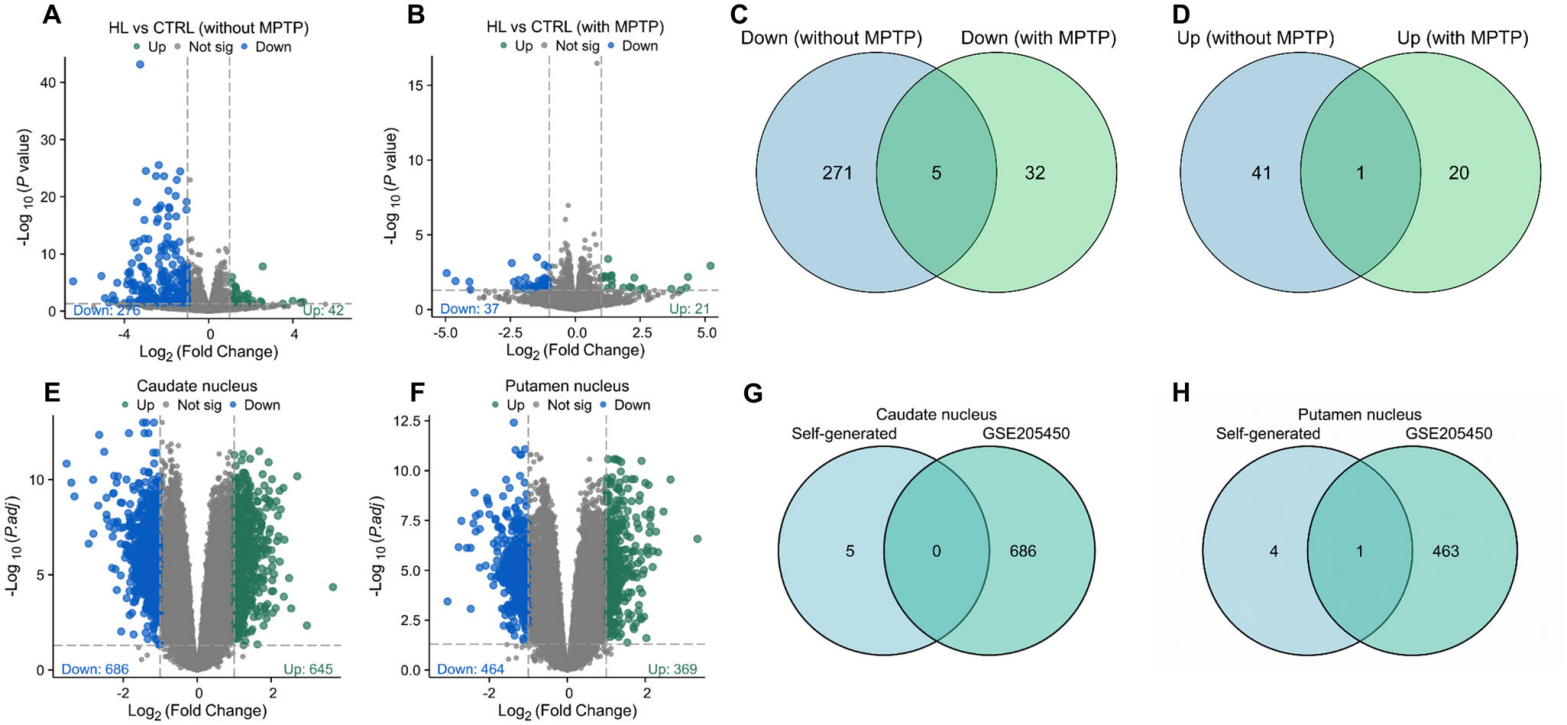
Cross‐species transcriptomic identification of Adcyap1 as a candidate molecular link between auditory deprivation and PD‐related alterations. (A) Volcano plot showing differentially expressed genes (DEGs) in the striatum of auditory‐deprived mice compared with control mice in the absence of MPTP exposure. (B) Volcano plot showing DEGs in the striatum of auditory‐deprived mice compared with MPTP‐only mice under MPTP exposure. (C) Venn diagram showing the overlap of downregulated genes between the two mouse comparisons shown in panels A and B. (D) Venn diagram showing the overlap of upregulated genes between the two mouse comparisons shown in panels A and B. (E) Volcano plot of differential gene expression in the human caudate nucleus from the GSE205450 dataset comparing PD and control donors. (F) Volcano plot of differential gene expression in the human putamen nucleus from the GSE205450 dataset comparing PD and control donors. (G) Venn diagram showing the overlap between candidate auditory‐deprivation‐associated mouse genes and DEGs identified in the human caudate nucleus. (H) Venn diagram showing the overlap between candidate auditory‐deprivation‐associated mouse genes and DEGs identified in the human putamen nucleus. Adcyap1 was the only overlapping gene in the putamen analysis and was therefore prioritized for subsequent investigation. In the mouse RNA‐seq analyses, DEGs were defined as genes with |log2 fold change| > 1 and *p* < 0.05 using DESeq2. In the human GSE205450 analysis, differential expression was evaluated using limma with voomWithQualityWeights and Benjamini–Hochberg correction. Upregulated genes are shown in green, downregulated genes in blue, and non‐significant genes in gray.

**TABLE 2:** Candidate genes consistently altered by auditory deprivation under MPTP and non‐MPTP conditions.

Group	Gene	HL1	HL2	HL3	Con1	Con2	Con3	log2FC	*p* value
MPTP	Adcyap1	0.211	0.092	0.028	0.138	0.259	0.374	−1.224	0.03834
MPTP	Endou	0.073	0.137	0.027	0.115	0.242	0.240	−1.322	0.04511
MPTP	Gmnc	0.050	0.089	0.066	0.204	0.185	0.108	−1.258	0.01593
MPTP	Mybpc1	0.043	0.041	0.012	0.086	0.074	0.141	−1.646	0.02852
MPTP	Pappa2	0.025	0.018	0.033	0.053	0.043	0.104	−1.392	0.02067
MPTP	Gm6710	0.141	0.119	0.479	0.141	0.027	0.114	1.409	0.02540
Non‐MPTP	Adcyap1	0.119	0.074	0.166	1.641	0.451	0.029	−2.578	0.04580
Non‐MPTP	Endou	0.105	0.085	0.074	0.395	0.159	0.132	−1.386	0.02955
Non‐MPTP	Gmnc	0.075	0.063	0.062	0.421	0.691	0.338	−2.870	2.64000E‐11
Non‐MPTP	Mybpc1	0.013	0.033	0.032	0.347	0.103	0.104	−2.837	0.00021
Non‐MPTP	Pappa2	0.029	0.011	0.034	0.126	0.062	0.028	−1.558	0.02586
Non‐MPTP	Gm6710	0.635	0.717	0.295	0.097	0.106	0.072	2.570	1.56000E‐08

*Note*: HL1–HL3 and Con1–Con3 represent normalized expression values from three independent biological replicates per condition. For the MPTP group, HL1–HL3 correspond to HL+MPTP mice and Con1–Con3 correspond to MPTP‐only mice; for the non‐MPTP group, HL1–HL3 correspond to auditory‐deprived mice and Con1–Con3 correspond to untreated controls. Differential‐expression testing was performed on raw gene‐level count data using DESeq2. Log_2_ fold change (log_2_FC) represents the expression change associated with auditory deprivation relative to the corresponding control condition (HL+MPTP vs. MPTP for the MPTP group and HL vs. Control for the non‐MPTP group). The six genes were retained as candidate auditory‐deprivation‐associated genes. Gm6710 is currently annotated as “predicted gene 6710” (MGI:3779623; Ensembl: ENSMUSG00000078887) in the GRCm39 mouse genome assembly.

### 3.3 Functional Enrichment Analysis of Adcyap1 and Its Interacting Proteins

Analysis using the GeneMANIA platform identified a network of genes potentially interacting with Adcyap1, suggesting that the proteins encoded by those genes may cooperate in executing related biological processes. GO and KEGG pathway enrichment analyses of Adcyap1 and its interacting proteins revealed (Figures 5A‐B) that these genes are predominantly involved in G protein–coupled receptor–related signaling processes and neuropeptide‐mediated communication. The top enriched biological processes included adenylate cyclase–modulating and cAMP‐mediated signaling pathways, peptide and hormone secretion, and the positive regulation of cyclic nucleotide signaling. Cellular component analysis indicated localization primarily within neuronal cell bodies, perikarya, and membrane microdomains such as lipid rafts—structures crucial for synaptic signaling and receptor trafficking. Molecular function terms were enriched for hormone activity, neuropeptide receptor binding, and adenylate cyclase interaction, underscoring the role of Adcyap1 in neuroendocrine and neurotransmission regulation. Consistent with these findings, KEGG pathway analysis highlighted significant enrichment in the “Neuroactive ligand–receptor interaction” and “cAMP signaling pathway,” suggesting that Adcyap1 and its network partners modulate neuronal excitability and intracellular signaling cascades relevant to dopaminergic function and neurodegeneration.

**FIGURE 5:**
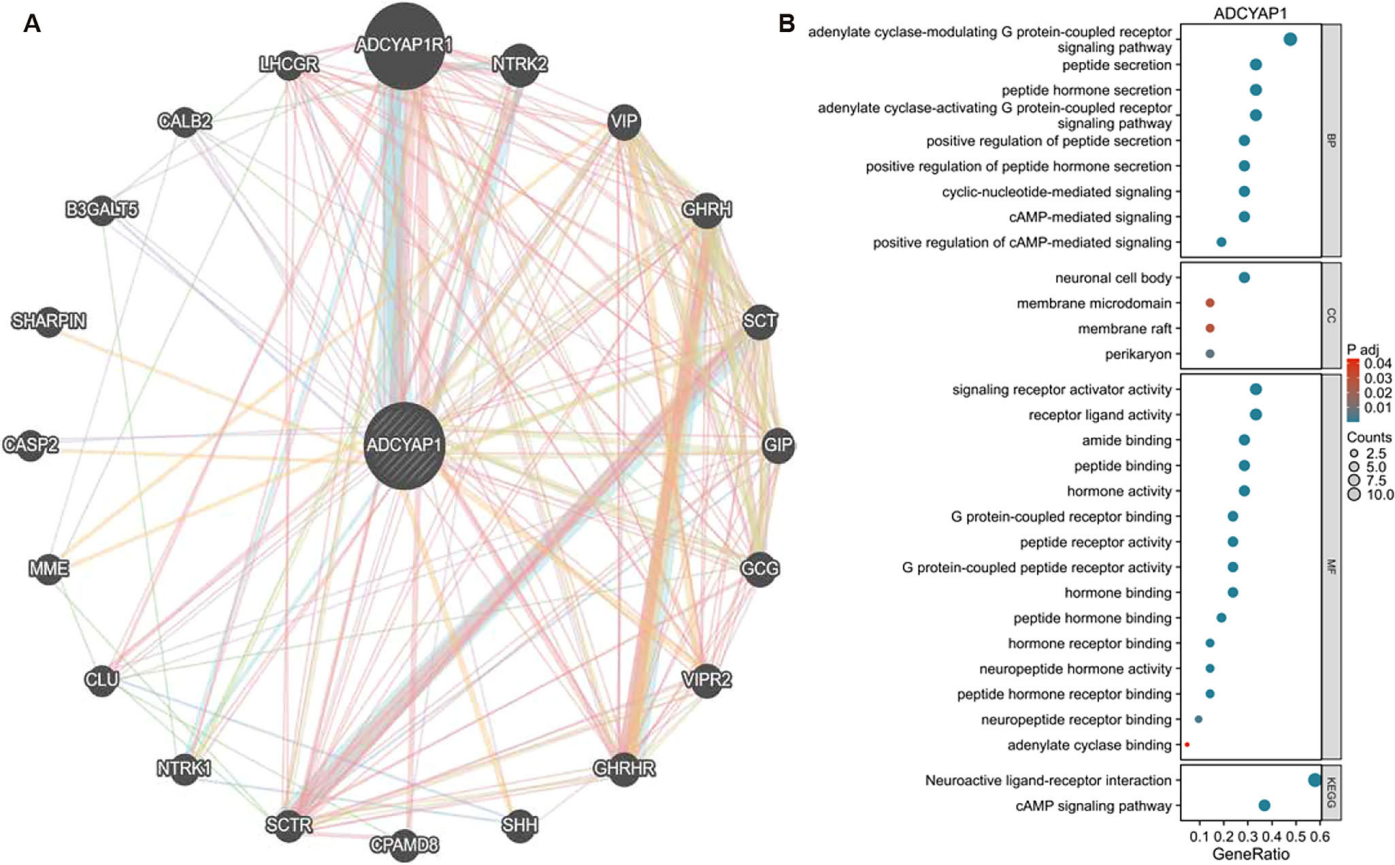
Functional network and pathway enrichment analysis of Adcyap1 implicates neuropeptide and cAMP signaling pathways. (A) Protein–protein interaction network of Adcyap1 and its predicted interacting genes generated using GeneMANIA. (B) Combined GO enrichment analyses (BP, CC, and MF) and KEGG pathway enrichment analysis of ADCYAP1‐associated genes. Dot size represents gene count and color represents adjusted *p* value.

## 4 Discussion

This study integrates complementary epidemiological, experimental, and molecular evidence regarding the relationship between hearing impairment and PD. Analyses of two independent population databases demonstrated consistent positive associations after adjustment for potential confounders and PSM. In mice, significant interactions between auditory deprivation and MPTP exposure were observed for motor performance and dopaminergic neuronal loss. Transcriptomic profiling further identified Adcyap1 downregulation in auditory‐deprived mice, with a concordant alteration observed in human PD striatal data. Together, these findings identify Adcyap1/PACAP signaling as a candidate molecular link that warrants functional validation.

Our epidemiological results align with prior large‐cohort studies reporting an association between HL and PD (Simonet et al. 2022; Neilson et al. 2024). Although some observational studies have reported null findings (Readman et al. 2023), the broadly consistent associations observed across two independent populations in the present study support further investigation of HL as a potential marker of PD‐related vulnerability.

Notably, Ning et al. reported no evidence that genetically predicted hearing loss causally increases PD risk in a two‐sample Mendelian randomization analysis (Ning et al. 2024). This finding does not necessarily conflict with the associations observed in the present study because the two approaches address different questions. Mendelian randomization estimates the effect of lifelong genetically proxied susceptibility to hearing loss, whereas our population analyses evaluate phenotypic associations between self‐reported hearing impairment and PD, and our animal experiment examines whether experimentally reduced auditory input modifies MPTP‐related phenotypes. Differences in hearing‐loss phenotype, severity, timing of exposure, and population characteristics may therefore contribute to the divergent findings, as also suggested by a recent triangulation analysis combining observational and Mendelian‐randomization evidence (Zhang et al. 2025). Accordingly, our results should not be interpreted as establishing HL as a causal risk factor for PD; rather, they provide epidemiological and experimental evidence of an association that requires further longitudinal and genetically informed investigation.

The experimental findings provide biological support for the epidemiological association. Factorial analyses demonstrated significant HL × MPTP interactions for motor performance and TH‐positive neuron counts. HL+MPTP mice showed poorer motor performance and substantially fewer TH‐positive neurons than MPTP‐only mice, whereas auditory deprivation alone was not associated with significant motor or dopaminergic changes after multiple‐comparison correction. The use of ossicular chain interruption in the present study also serves a specific experimental purpose. Age‐related sensorineural hearing loss involves not only reduced auditory input but also degeneration of cochlear sensory and neural structures and age‐associated processes such as oxidative stress, mitochondrial dysfunction, and inflammation (Yang et al. 2023). These processes may overlap with broader mechanisms of neural aging and neurodegeneration, making it difficult to distinguish the consequences of auditory deprivation itself from those associated with concomitant peripheral degeneration. In contrast, ossicular chain interruption markedly reduces acoustic input without intentionally inducing cochlear sensory injury, thereby providing a useful paradigm for more specifically examining the biological consequences associated with reduced auditory input. Conductive auditory deprivation has been shown to alter central auditory processing while relatively preserving peripheral cochlear function (Xu et al. 2007, Ye et al. 2021). Thus, rather than attempting to reproduce the predominant clinical etiology of hearing loss in older adults, this model allows us to examine whether auditory deprivation itself modifies MPTP‐related phenotypes. Nevertheless, its relevance to age‐related sensorineural hearing loss requires confirmation in complementary sensorineural models.

Mechanistically, transcriptomic analyses identified reduced striatal Adcyap1 expression following auditory deprivation, thereby prioritizing Adcyap1/PACAP signaling as a candidate molecular link. PACAP signaling itself has previously been implicated independently in both auditory biology and PD. PACAP and its PAC1 receptor are present in the cochlea and participate in auditory signaling, and PACAP deficiency is associated with hearing impairment (Drescher et al. 2006, Fulop et al. 2019). In experimental PD, PACAP has shown neuroprotective effects in MPTP‐treated mice (Wang et al. 2008), while reduced Adcyap1/PAC1 receptor expression has been reported in the caudate‐putamen and substantia nigra in a rotenone model (Fehér et al. 2023). Thus, the potentially novel aspect of our findings is not the involvement of PACAP in hearing or PD per se, but the observation that conductive auditory deprivation was associated with striatal Adcyap1 downregulation both in the absence and presence of MPTP exposure, with directionally concordant downregulation also identified in human PD putamen. Because ossicular chain interruption reduces auditory input without intentionally inducing cochlear sensory injury, this finding raises the possibility that altered central Adcyap1/PACAP signaling may represent a molecular response associated with auditory deprivation itself. This interpretation remains hypothesis‐generating, and direct manipulation of Adcyap1/PACAP signaling will be required to establish causality.

Clinically, our findings carry important implications. HL is common, clinically detectable, and potentially modifiable, making its association with PD clinically relevant and worthy of further longitudinal investigation. Early hearing assessment and rehabilitation—via hearing aids, cochlear implants, or auditory training—could potentially restore sensory input, stabilize network connectivity, and reduce central inflammatory burden. Epidemiological evidence showing lower PD incidence among hearing aid users further supports this hypothesis (Neilson et al. 2024), though randomized trials are needed to confirm causality. From a therapeutic perspective, modulation of the Adcyap1/PACAP pathway represents an attractive neuroprotective strategy. Whether pharmacologic modulation of PACAP signaling or Adcyap1 expression can modify dopaminergic vulnerability in the context of auditory deprivation requires direct functional investigation. Integration of PACAP‐related biomarkers into PD risk prediction frameworks could also improve early identification and targeted prevention.

Despite these strengths, certain limitations should be acknowledged. The population analyses relied on self‐reported hearing status and cohort‐specific proxy ascertainment of PD. In CHARLS, PD was based on self‐reported physician diagnosis, whereas in NHANES it was operationally ascertained from self‐reported anti‐Parkinsonian medication use. These measures may introduce exposure or outcome misclassification, and the observational design limits causal inference. The conductive auditory‐deprivation model was selected to reduce auditory input without intentionally inducing cochlear sensory degeneration, thereby facilitating examination of the consequences associated with auditory deprivation itself. However, because this model does not reproduce the cochlear pathology of age‐related sensorineural hearing loss, the present findings should not be directly generalized to the predominant forms of hearing loss in older adults. Replication in age‐related and noise‐induced sensorineural hearing‐loss models will therefore be important. In addition, the MPTP paradigm models toxin‐induced dopaminergic injury rather than progressive α‐synucleinopathy. Future studies should determine whether the present findings can be reproduced in noise‐induced and age‐related sensorineural hearing‐loss models. Importantly, the present study did not include functional manipulation of Adcyap1/PACAP signaling. Therefore, the observed cross‐species downregulation cannot determine whether Adcyap1 is causally involved, represents a downstream consequence, or reflects a correlated molecular response. Rescue or gain‐of‐function experiments, such as PACAP administration or Adcyap1 overexpression, will be required to establish whether this pathway functionally contributes to the observed auditory deprivation–MPTP interaction. Finally, the total number of MPTP treatment blocks was determined adaptively on the basis of interim behavioral performance rather than by a pre‐specified stopping rule, which should be considered when interpreting the animal‐model procedure.

In a broader context, this work contributes to the emerging concept of “sensory reserve,” whereby preservation of sensory input supports neural resilience, and sensory deprivation has been associated with reduced neural resilience. Recognizing auditory dysfunction as part of the neurodegenerative cascade reframes PD as a multisystem disorder involving the interaction between sensory, cognitive, and motor networks.

## 5 Conclusion

Our findings show consistent associations between self‐reported hearing impairment and PD across two population datasets. In mice, auditory deprivation interacted with MPTP exposure in motor and dopaminergic phenotypes, providing experimental support for a potential biological relationship. Cross‐species transcriptomic analyses further identified Adcyap1/PACAP signaling as a candidate molecular link between auditory deprivation and PD‐related alterations. Functional studies are required to determine whether this pathway plays a causal role in the observed relationship.

## Author Contributions

**Xiao Yu**: formal analysis, software, visualization, writing – original draft, conceptualization, methodology, project administration. **Wenxin Shen**: visualization, writing – review and editing. **Jiayu Li**: writing – review and editing, data curation. **Yiming Ma**: writing – review and editing, data curation. **Jingjing Cai**: data curation, writing – review and editing. **Jian Wang**: writing – review and editing, visualization. **Hui Wang**: conceptualization, funding acquisition, writing – review and editing, methodology, project administration. **Shankai Yin**: conceptualization, writing – review and editing. All authors critically revised the manuscript for intellectual content, approved the final version, and agree to be accountable for all aspects of the work.

## Funding

This work was supported by the National Key R&D Program (2024YFC2418100), Shanghai Oriental Talents Excellent Academic Leader Program (dfyc2023), and Two‐Hundred Talent of Shanghai Jiao Tong University School of Medicine (SBR2022001).

## Ethics Statement

The human population analyses were based on de‐identified data from the National Health and Nutrition Examination Survey (NHANES) and the China Health and Retirement Longitudinal Study (CHARLS). Ethical approval and informed consent were obtained by the original survey organizers, and no additional individual consent was required for the present secondary analysis. All animal experimental procedures were performed in accordance with the National Institutes of Health Guidelines for the Care and Use of Laboratory Animals and were approved by the Laboratory Animal Center of Shanghai Jiao Tong University (approval number: A2024333‐001). All efforts were made to minimize animal suffering and to reduce the number of animals used.

## Consent

The authors have nothing to report.

## Conflicts of Interest

The authors declare no conflicts of interest.

## Supporting information




**Supplementary Material**: brb371806‐sup‐0001‐SuppMat.docx

## Data Availability

The NHANES and CHARLS datasets analyzed in this study are publicly available from their respective official repositories. The human postmortem transcriptomic dataset GSE205450 is publicly available in the Gene Expression Omnibus (GEO). The mouse striatal RNA‐sequencing data generated in the present study have been deposited in Figshare and are publicly available at https://doi.org/10.6084/m9.figshare.33264708. Additional derived data are available from the corresponding author upon reasonable request.

## References

[brb371806-bib-0001] Armstrong, M. J. , and M. S. Okun . 2020. “Diagnosis and Treatment of Parkinson Disease.” Jama 323, no. 6: 548. 10.1001/jama.2019.22360. 32044947

[brb371806-bib-0002] Bloem, B. R. , M. S. Okun , and C. Klein . 2021. “Parkinson's Disease.” The Lancet 397, no. 10291: 2284–2303. 10.1016/S0140-6736(21)00218-X. 33848468

[brb371806-bib-0003] Chen, J. J. 2010. “Parkinson's Disease: Health‐Related Quality of Life, Economic Cost, and Implications of Early Treatment.” American Journal of Managed Care 16: S87–S93. 20297871

[brb371806-bib-0004] Chen, L. , X. Wang , S. Ge , and Q. Xiong . 2019. “Medial Geniculate Body and Primary Auditory Cortex Differentially Contribute to Striatal Sound Representations.” Nature Communications 10, no. 1: 418. 10.1038/s41467-019-08350-7. PMC6346050 30679433

[brb371806-bib-0005] Chen, Z. , H. Xie , L. Yao , and Y. Wei . 2021. “Olfactory Impairment and the Risk of Cognitive Decline and Dementia in Older Adults: A Meta‐Analysis.” Brazilian Journal of Otorhinolaryngology 87, no. 1: 94–102. 10.1016/j.bjorl.2020.07.009. PMC9422530 32978117

[brb371806-bib-0006] Daalen, J. M. J. , S. Schootemeijer , E. Richard , S. K. L. Darweesh , and B. R. Bloem . 2022. “Lifestyle Interventions for the Prevention of Parkinson Disease.” Neurology 99, no. 7: 42–51. 10.1212/WNL.0000000000200787. 35970584

[brb371806-bib-0007] Dintica, C. S. , A. Marseglia , D. Rizzuto , et al. 2019. “Impaired Olfaction is Associated With Cognitive Decline and Neurodegeneration in the Brain.” Neurology 92, no. 7: e700–e709. 10.1212/WNL.0000000000006919. PMC6382360 30651382

[brb371806-bib-0008] Drescher, M. J. , D. G. Drescher , K. M. Khan , et al. 2006. “Pituitary Adenylyl Cyclase‐Activating Polypeptide (PACAP) and Its Receptor (PAC1‐R) are Positioned to Modulate Afferent Signaling in the Cochlea.” Neuroscience 142, no. 1: 139–164. 10.1016/j.neuroscience.2006.05.065. 16876955

[brb371806-bib-0009] Fehér, M. , Z. Márton , Á. Szabó , et al. 2023. “Downregulation of PACAP and the PAC1 Receptor in the Basal Ganglia, Substantia Nigra and Centrally Projecting Edinger‐Westphal Nucleus in the Rotenone Model of Parkinson's Disease.” International Journal of Molecular Sciences 24, no. 14: 11843. 10.3390/ijms241411843 PMC10380602 37511603

[brb371806-bib-0010] Ferraz, H. B. , and C. C. Silva . 2016. “Unusual Early Symptoms of Parkinson's Disease. Why Do We Need to Identify Them?” Arquivos de Neuro‐Psiquiatria 74, no. 10: 779–780. 10.1590/0004-282X20160135. 27759800

[brb371806-bib-0011] Fulop, D. B. , V. Humli , J. Szepesy , et al. 2019. “Hearing Impairment and Associated Morphological Changes in Pituitary Adenylate Cyclase Activating Polypeptide (PACAP)‐Deficient Mice.” Scientific Reports 9, no. 1: 14598. 10.1038/s41598-019-50775-z. PMC6787024 31601840

[brb371806-bib-0012] Gammon, K. 2014. “Neurodegenerative Disease: Brain Windfall.” Nature 515, no. 7526: 299–300. 10.1038/nj7526-299a. 25396246

[brb371806-bib-0013] GBD 2015 Neurological Disorders Collaborator Group . 2017. “Global, Regional, and National Burden of Neurological Disorders During 1990–2015: A Systematic Analysis for the Global Burden of Disease Study 2015.” The Lancet Neurology 16, no. 11: 877–897. 10.1016/S1474-4422(17)30299-5. PMC5641502 28931491

[brb371806-bib-0014] GBD 2016 Parkinson's Disease Collaborators . 2018. “Global, Regional, and National Burden of Parkinson's Disease, 1990–2016: A Systematic Analysis for the Global Burden of Disease Study 2016.” The Lancet Neurology 17, no. 11: 939–953. 10.1016/S1474-4422(18)30295-3. PMC6191528 30287051

[brb371806-bib-0015] Hu, M. , C. Skjærbæk , and P. Borghammer . 2024. “Approaches to Early Parkinson's Disease Subtyping.” Journal of Parkinson's Disease 14, no. S2: S297–S306. 10.3233/JPD-230419. PMC11492007 39331104

[brb371806-bib-0016] Irmady, K. , C. R. Hale , R. Qadri , et al. 2023. “Blood Transcriptomic Signatures Associated With Molecular Changes in the Brain and Clinical Outcomes in Parkinson's Disease.” Nature Communications 14, no. 1: 3956. 10.1038/s41467-023-39652-6. PMC10322907 37407548

[brb371806-bib-0017] Jafari, Z. , B. E. Kolb , and M. H. Mohajerani . 2020. “Auditory Dysfunction in Parkinson's Disease.” Movement Disorders 35, no. 4: 537–550. 10.1002/mds.28000. 32052894

[brb371806-bib-0018] Lai, S. W. , K. F. Liao , C. L. Lin , C. C. Lin , and F. C. Sung . 2014. “Hearing Loss May be a Non‐Motor Feature of Parkinson's Disease in Older People in Taiwan.” European Journal of Neurology 21, no. 5: 752–757. 10.1111/ene.12378. 24506292

[brb371806-bib-0019] Leme, M. S. , S. G. G. Sanches , and R. M. M. Carvallo . 2023. “Peripheral Hearing in Parkinson's Disease: A Systematic Review.” International Journal of Audiology 62, no. 9: 805–813. 10.1080/14992027.2022.2109073. 35980314

[brb371806-bib-0020] Li, S. , C. Cheng , L. Lu , et al. 2021. “Hearing Loss in Neurological Disorders.” Frontiers in Cell and Developmental Biology 9: 716300. 10.3389/fcell.2021.716300. PMC8385440 34458270

[brb371806-bib-0021] Lotankar, S. , K. S. Prabhavalkar , and L. K. Bhatt . 2017. “Biomarkers for Parkinson's Disease: Recent Advancement.” Neuroscience Bulletin 33, no. 5: 585–597. 10.1007/s12264-017-0183-5. PMC5636742 28936761

[brb371806-bib-0022] Montgomery Jr, E. B. , A. Lieberman , G. Singh , and J. F. Fries . 1994. “Patient Education and Health Promotion Can be Effective in Parkinson's Disease: A Randomized Controlled Trial. PROPATH Advisory Board.” American Journal of Medicine 97, no. 5: 429–435. 10.1016/0002-9343(94)90322-0 7977431

[brb371806-bib-0023] Mowery, T. M. , K. B. Penikis , S. K. Young , C. E. Ferrer , V. C. Kotak , and D. H. Sanes . 2017. “The Sensory Striatum is Permanently Impaired by Transient Developmental Deprivation.” Cell Reports 19, no. 12: 2462–2468. 10.1016/j.celrep.2017.05.083. PMC5577933 28636935

[brb371806-bib-0024] Neilson, L. E. , K. M. Reavis , J. Wiedrick , and G. D. Scott . 2024. “Hearing Loss, Incident Parkinson Disease, and Treatment With Hearing Aids.” JAMA Neurology 81, no. 12: 1295. 10.1001/jamaneurol.2024.3568. PMC11581660 39432289

[brb371806-bib-0025] Ning, P. , X. Mu , X. Guo , and R. Li . 2024. “Hearing Loss is not Associated With Risk of Parkinson's Disease: A Mendelian Randomization Study.” Heliyon 10, no. 11: e32533. 10.1016/j.heliyon.2024.e32533. PMC11219492 38961984

[brb371806-bib-0026] Ou, C. , B. Chen , H. Yu , J. Deng , and H. Long . 2026. “Association Between the Triglyceride‐Glucose Frailty Index and Parkinson's Disease in Middle‐Aged and Older Adults: A Comparative Cross‐National Analysis of NHANES and CHARLS.” Parkinsonism & Related Disorders 145: 108240. 10.1016/j.parkreldis.2026.108240. 41691976

[brb371806-bib-0027] Paulsen, A. J. , C. R. Schubert , A. A. Pinto , et al. 2022. “Associations of Sensory and Motor Function With Blood‐Based Biomarkers of Neurodegeneration and Alzheimer's Disease in Midlife.” Neurobiology of Aging 120: 177–188. 10.1016/j.neurobiolaging.2022.08.008. PMC9613601 36209638

[brb371806-bib-0028] Readman, M. R. , F. Wan , I. Fairman , S. A. Linkenauger , T. J. Crawford , and C. J. Plack . 2023. “Is Hearing Loss a Risk Factor for Idiopathic Parkinson's Disease? An English Longitudinal Study of Ageing Analysis.” Brain Sciences 13, no. 8: 1196. 10.3390/brainsci13081196. PMC10452744 37626551

[brb371806-bib-0029] Simonet, C. , J. Bestwick , M. Jitlal , et al. 2022. “Assessment of Risk Factors and Early Presentations of Parkinson Disease in Primary Care in a Diverse UK Population.” JAMA Neurology 79, no. 4: 359. 10.1001/jamaneurol.2022.0003. PMC8902684 35254398

[brb371806-bib-0030] Su, D. , Y. Cui , C. He , et al. 2025. “Projections for Prevalence of Parkinson's Disease and Its Driving Factors in 195 Countries and territories to 2050: Modelling Study of Global Burden of Disease Study 2021.” Bmj 388: e080952. 10.1136/bmj-2024-080952. PMC11881235 40044233

[brb371806-bib-0031] Troshev, D. , A. Bannikova , V. Blokhin , E. Pavlova , A. Kolacheva , and M. Ugrumov . 2023. “Compensatory Processes in Striatal Neurons Expressing the Tyrosine Hydroxylase Gene in Transgenic Mice in a Model of Parkinson's Disease.” International Journal of Molecular Sciences 24, no. 22: 16245. 10.3390/ijms242216245. PMC10671746 38003434

[brb371806-bib-0032] Tu, X. , N. Wu , Y. Wan , J. Gan , Z. Liu , and L. Song . 2024. “Association of Dietary Selenium Intake and All‐Cause Mortality of Parkinson's Disease and Its Interaction With Blood Cadmium Level: A Retrospective Cohort Study.” BMC Geriatrics 24, no. 1: 415. 10.1186/s12877-024-05000-6. PMC11088170 38730347

[brb371806-bib-0033] van der Berg, I. , S. Schootemeijer , K. Overbeek , B. R. Bloem , and N. M. de Vries . 2024. “Dietary Interventions in Parkinson's Disease.” Journal of Parkinsons Disesase 14, no. 1: 1–16. 10.3233/JPD-230366 PMC10836553 38277304

[brb371806-bib-0034] Wang, G. , J. Pan , Y.u‐Y. Tan , et al. 2008. “Neuroprotective Effects of PACAP27 in Mice Model of Parkinson's Disease Involved in the Modulation of KATP Subunits and D2 Receptors in the Striatum.” Neuropeptides 42, no. 3: 267–276. 10.1016/j.npep.2008.03.002. 18440632

[brb371806-bib-0035] Wang, Y.u , Y. Xie , M. Wang , et al. 2025. “Hearing Loss Prevalence and Burden of Disease in China: Findings From Provincial‐Level Analysis.” Chinese Medical Journal 138, no. 1: 41–48. 10.1097/CM9.0000000000003096. PMC11717509 38725346

[brb371806-bib-0036] Xu, H. , V. C. Kotak , and D. H. Sanes . 2007. “Conductive Hearing Loss Disrupts Synaptic and Spike Adaptation in Developing Auditory Cortex.” The Journal of Neuroscience 27, no. 35: 9417–9426. 10.1523/JNEUROSCI.1992-07.2007. PMC6673134 17728455

[brb371806-bib-0037] Xu, T. , W. Dong , J. Liu , et al. 2024. “Disease Burden of Parkinson's Disease in China and Its Provinces From 1990 to 2021: Findings From the Global Burden of Disease Study 2021.” The Lancet Regional Health–Western Pacific 46: 101078. 10.1016/j.lanwpc.2024.101078 PMC11091691 38745974

[brb371806-bib-0038] Yang, W. , J. L. Hamilton , C. Kopil , et al. 2020. “Current and Projected Future Economic Burden of Parkinson's Disease in the U.S.” npj Parkinson's Disease 6: 15. 10.1038/s41531-020-0117-1. PMC7347582 32665974

[brb371806-bib-0039] Yang, W. , X. Zhao , R. Chai , and J. Fan . 2023. “Progress on Mechanisms of Age‐Related Hearing Loss.” Frontiers in Neuroscience 17: 1253574. 10.3389/fnins.2023.1253574. PMC10505809 37727326

[brb371806-bib-0040] Yao, C. W. , A. Szpindel , A. Pelletier , and R. B. Postuma . 2023. “Hearing Impairment and Development of Parkinsonism and Possible Rapid Eye Movement Sleep Behaviour Disorder: A CLSA Prospective Population‐Based Study.” European Journal of Neurology 30, no. 1: 287–289. 10.1111/ene.15567. 36103188

[brb371806-bib-0041] Ye, Y. , A. Ihlefeld , and M. J. Rosen . 2021. “Conductive Hearing Loss During Development Does Not Appreciably Alter the Sharpness of Cochlear Tuning.” Scientific Reports 11, no. 1: 3955. 10.1038/s41598-021-83115-1. PMC7890061 33597563

[brb371806-bib-0042] Zhang, H. , K. Chen , T. Gao , et al. 2025. “Establishing a Robust Triangulation Framework to Explore the Relationship Between Hearing Loss and Parkinson's Disease.” npj Parkinson's Disease 11, no. 1: 5. 10.1038/s41531-024-00861-5. PMC11698951 39753591

[brb371806-bib-0043] Zhao, Y. , D. Luo , Z. Ning , J. Rong , and L. Lao . 2019. “Electro‐Acupuncture Ameliorated MPTP‐Induced Parkinsonism in Mice via TrkB Neurotrophic Signaling.” Frontiers in Neuroscience 13: 496. 10.3389/fnins.2019.00496. PMC6528026 31156376

