# Supplementary material for: Hearing Loss and Parkinson's Disease: Insights From Human and Animal Studies
Source: Brain Behav. 2026 Oct 21;16(10):e71806. doi: 10.1002/brb3.71806 (PMC13687657; doi:10.1002/brb3.71806)
Supplement: Supplementary file 1 [file BRB3-16-e71806-s001.docx]

**Supplementary Material**

**Table S1. Baseline characteristics of the subjects analyzed in NHANES.**

| **Variables from NHANES** | **Control (n=17517)** | **PD (n=266)** | ***P* value** |
| --- | --- | --- | --- |
| Age, n(%) |  |  | 0.28 |
| ≤ 55 | 5675 (40.46) | 69 (35.17) |  |
| > 55 | 11842 (59.54) | 197 (64.83) |  |
| Sex, n(%) |  |  | 0.07 |
| Female | 8902 (53.03) | 136 (61.42) |  |
| Male | 8615 (46.97) | 130 (38.58) |  |
| Race, n(%) |  |  | 0.002 |
| Mexican American | 2408 (5.39) | 23 (3.13) |  |
| Non-Hispanic Black | 3919 (10.41) | 37 (7.48) |  |
| Non-Hispanic White | 7781 (73.00) | 174 (82.75) |  |
| Other Race | 3409 (11.20) | 32 (6.63) |  |
| Marital, n(%) |  |  | 0.08 |
| never married | 1315 (6.43) | 29 (9.05) |  |
| widowed/separated/divorced | 5673 (27.08) | 90 (32.51) |  |
| married/like married | 10513 (66.49) | 147 (58.44) |  |
| Education, n(%) |  |  | 0.24 |
| less than high school | 5076 (17.11) | 89 (21.94) |  |
| high school | 4040 (24.46) | 59 (26.47) |  |
| more than high school | 8370 (58.43) | 118 (51.59) |  |
| BMI, n(%) |  |  | 0.12 |
| normal | 4135 (26.07) | 58 (28.91) |  |
| overweight | 5754 (35.12) | 66 (26.03) |  |
| obese | 6486 (38.82) | 113 (45.06) |  |
| Smoker, n(%) |  |  | 0.09 |
| never | 8943 (51.52) | 124 (50.03) |  |
| former | 5491 (31.76) | 88 (27.31) |  |
| now | 3069 (16.73) | 53 (22.66) |  |
| HL, n(%) |  |  | <0.001 |
| no | 11775 (68.48) | 127 (52.57) |  |
| yes | 5742 (31.52) | 139 (47.43) |  |
| Hearing aid use, n(%) |  |  | 0.02 |
| no | 2515 (66.67) | 43 (49.43) |  |
| yes | 1001 (33.33) | 42 (50.57) |  |
| Diabetes, n(%) |  |  | 0.03 |
| no | 14059 (85.27) | 195 (79.36) |  |
| yes | 3443 (14.73) | 71 (20.64) |  |
| Coronary heart disease, n(%) |  |  | 0.001 |
| no | 16236 (94.12) | 225 (87.68) |  |
| yes | 1173 (5.88) | 38 (12.32) |  |
| Hypertension, n(%) |  |  | 0.001 |
| no | 8587 (54.52) | 98 (41.22) |  |
| yes | 8900 (45.48) | 168 (58.78) |  |
| Stroke, n(%) |  |  | <0.001 |
| no | 16325 (95.01) | 219 (83.03) |  |
| yes | 1155 (4.99) | 47 (16.97) |  |
| Hyperlipidemia, n(%) |  |  | 0.99 |
| no | 3633 (20.63) | 47 (20.59) |  |
| yes | 13078 (79.37) | 205 (79.41) |  |
| Cancer, n(%) |  |  | 0.09 |
| no | 14933 (84.13) | 213 (78.18) |  |
| yes | 2559 (15.87) | 51 (21.82) |  |

Values are presented as unweighted counts and survey-weighted percentages. Therefore, weighted percentages may not correspond directly to the proportions obtained by dividing the reported counts by the total sample size.

**Table S2. Baseline characteristics of the subjects analyzed in CHARLS.**

| **Variables from CHARLS** | **Control (n = 17482)** | **PD (n = 181)** | ***P* value** |
| --- | --- | --- | --- |
| Age, n(%) |  |  | <0.001 |
| ≤ 55 | 4325 (24.74) | 7 (3.87) |  |
| > 55 | 13157 (75.26) | 174 (96.13) |  |
| Sex, n(%) |  |  | 0.992 |
| female | 9279 (53.08) | 96 (53.04) |  |
| male | 8203 (46.92) | 85 (46.96) |  |
| Marital, n(%) |  |  | 0.001 |
| never married | 104 (0.59) | 1 (0.55) |  |
| widowed/separated/divorced | 2707 (15.48) | 46 (25.41) |  |
| married | 14671 (83.92) | 134 (74.03) |  |
| Education, n(%) |  |  | <0.001 |
| less than high school | 15340 (87.75) | 156 (86.19) |  |
| high school | 1809 (10.35) | 14 (7.73) |  |
| more than high school | 333 (1.90) | 11 (6.08) |  |
| Smoker, n(%) |  |  | <0.001 |
| never | 10700 (61.24) | 104 (57.46) |  |
| former | 2329 (13.33) | 46 (25.41) |  |
| now | 4444 (25.43) | 31 (17.13) |  |
| HL, n(%) |  |  | <0.001 |
| no | 13905 (79.54) | 107 (59.12) |  |
| yes | 3577 (20.46) | 74 (40.88) |  |
| Hearing aid use, n(%) |  |  | 0.198 |
| no | 17094 (98.51) | 161 (96.99) |  |
| yes | 258 (1.49) | 5 (3.01) |  |
| Diabetes, n(%) |  |  | <0.001 |
| no | 13505 (77.52) | 107 (59.44) |  |
| yes | 3916 (22.48) | 73 (40.56) |  |
| Coronary heart disease, n(%) |  |  | <0.001 |
| no | 12527 (71.66) | 63 (35.00) |  |
| yes | 4953 (28.34) | 117 (65.00) |  |
| Hypertension, n(%) |  |  | <0.001 |
| no | 8217 (47.52) | 45 (25.42) |  |
| yes | 9075 (52.48) | 132 (74.58) |  |
| Stroke, n(%) |  |  | <0.001 |
| no | 15891 (91.12) | 113 (63.13) |  |
| yes | 1548 (8.88) | 66 (36.87) |  |
| Lipid disorder, n(%) |  |  | <0.001 |
| no | 9237 (53.22) | 55 (30.73) |  |
| yes | 8118 (46.78) | 124 (69.27) |  |
| Cancer, n(%) |  |  | 0.069 |
| no | 16903 (96.85) | 171 (94.48) |  |
| yes | 549 (3.15) | 10 (5.52) |  |

**Table S3. Baseline characteristics of the matched subjects in NHANES.**

| **Variables from NHANES** | **Control (n = 70)** | **PD (n = 70)** |
| --- | --- | --- |
| Sex, n(%) |  |  |
| female | 33 (47.14) | 32 (45.71) |
| male | 37 (52.86) | 38 (54.29) |
| Race, n(%) |  |  |
| mexican American | 3 (4.29) | 3 (4.29) |
| non-Hispanic Black | 5 (7.14) | 5 (7.14) |
| non-Hispanic White | 52 (74.29) | 53 (75.71) |
| other Race | 10 (14.29) | 9 (12.86) |
| Marital, n(%) |  |  |
| never married | 1 (1.43) | 1 (1.43) |
| widowed/separated/divorced | 25 (35.71) | 28 (40.00) |
| married/like married | 44 (62.86) | 41 (58.57) |
| Education, n(%) |  |  |
| less than high school | 27 (38.57) | 27 (38.57) |
| high school | 16 (22.86) | 17 (24.29) |
| more than high school | 27 (38.57) | 26 (37.14) |
| BMI, n(%) |  |  |
| normal | 22 (31.43) | 21 (30.00) |
| overweight | 26 (37.14) | 24 (34.29) |
| obese | 22 (31.43) | 25 (35.71) |
| Smoker, n(%) |  |  |
| former | 27 (38.57) | 27 (38.57) |
| never | 41 (58.57) | 41 (58.57) |
| now | 2 (2.86) | 2 (2.86) |
| HL, n(%) |  |  |
| no | 21 (30.00) | 11 (15.71) |
| yes | 49 (70.00) | 59 (84.29) |
| Hearing aid use, n(%) |  |  |
| no | 40 (57.14) | 33 (47.14) |
| yes | 30 (42.86) | 37 (52.86) |
| Diabetes, n(%) |  |  |
| no | 49 (70.00) | 48 (68.57) |
| yes | 21 (30.00) | 22 (31.43) |
| Coronary heart disease, n(%) |  |  |
| no | 58 (82.86) | 56 (80.00) |
| yes | 12 (17.14) | 14 (20.00) |
| Hypertension, n(%) |  |  |
| no | 20 (28.57) | 23 (32.86) |
| yes | 50 (71.43) | 47 (67.14) |
| Stroke, n(%) |  |  |
| no | 54 (77.14) | 55 (78.57) |
| yes | 16 (22.86) | 15 (21.43) |
| Hyperlipidemia, n(%) |  |  |
| no | 6 (8.57) | 8 (11.43) |
| yes | 64 (91.43) | 62 (88.57) |
| Cancer, n(%) |  |  |
| no | 53 (75.71) | 52 (74.29) |
| yes | 17 (24.29) | 18 (25.71) |

Values are presented as unweighted counts and percentages within the propensity score-matched samples. Covariate balance after matching was assessed using standardized mean differences and is reported in Supplementary Table S6.

**Table S4. Baseline characteristics of the matched subjects in CHARLS.**

| **Variables from CHARLS** | **Control (n = 171)** | **PD (n = 171)** |
| --- | --- | --- |
| Age, n(%) |  |  |
| ≤ 55 | 21 (12.28) | 22 (12.87) |
| > 55 | 150 (87.72) | 149 (87.13) |
| Sex, n(%) |  |  |
| female | 85 (49.71) | 93 (54.39) |
| male | 86 (50.29) | 78 (45.61) |
| Education, n(%) |  |  |
| less than high school | 153 (89.47) | 151 (88.30) |
| high school | 11 (6.43) | 13 (7.60) |
| more than high school | 7 (4.09) | 7 (4.09) |
| Marital, n(%) |  |  |
| never married | 2 (1.17) | 1 (0.58) |
| widowed/separated/divorced | 42 (24.56) | 43 (25.15) |
| married | 127 (74.27) | 127 (74.27) |
| HL, n(%) |  |  |
| no | 122 (71.35) | 103 (60.23) |
| yes | 49 (28.65) | 68 (39.77) |
| Hearing aid use, n(%) |  |  |
| no | 167 (98.82) | 152 (97.44) |
| yes | 2 (1.18) | 4 (2.56) |
| Smoker, n(%) |  |  |
| never | 100 (58.48) | 99 (57.89) |
| former | 42 (24.56) | 43 (25.15) |
| now | 29 (16.96) | 29 (16.96) |
| Cancer, n(%) |  |  |
| no | 167 (97.66) | 162 (94.74) |
| yes | 4 (2.34) | 9 (5.26) |
| Coronary heart disease, n(%) |  |  |
| no | 62 (36.26) | 62 (36.26) |
| yes | 109 (63.74) | 109 (63.74) |
| Diabetes, n(%) |  |  |
| no | 107 (62.57) | 104 (60.82) |
| yes | 64 (37.43) | 67 (39.18) |
| Lipid disorder, n(%) |  |  |
| no | 63 (37.28) | 51 (30.00) |
| yes | 106 (62.72) | 119 (70.00) |
| Hypertension, n(%) |  |  |
| no | 45 (26.32) | 44 (25.73) |
| yes | 126 (73.68) | 127 (74.27) |
| Stroke, n(%) |  |  |
| no | 113 (66.08) | 112 (65.50) |
| yes | 58 (33.92) | 59 (34.50) |

Values are presented as unweighted counts and percentages within the propensity score-matched samples. Covariate balance after matching was assessed using standardized mean differences and is reported in Supplementary Table S6.

**Table S5. Two-way ANOVA and Šidák-adjusted multiple comparisons for behavioral, ABR, and TH-positive neuron outcomes.**

**Table S5A. Behavioral outcomes**

| **Outcome** | **Assessment** | **HL main effect, F (P)** | **MPTP main effect, F (P)** | **HL × MPTP interaction, F (P)** | **Control vs HL, adjusted P** | **MPTP vs HL+MPTP, adjusted P** |
| --- | --- | --- | --- | --- | --- | --- |
| Open-field distance | Baseline | 0.71 (0.408) | 0.00 (0.974) | 0.93 (0.344) | 0.995 | 0.381 |
| Open-field distance | Test 1 | 3.62 (0.069) | 6.40 (0.018) | 1.84 (0.188) | 0.911 | 0.059 |
| Open-field distance | Test 2 | 13.80 (0.001) | 33.05 (<0.0001) | **5.23 (0.031)** | 0.541 | **0.0006** |
| Open-field distance | Test 3 | 34.39 (<0.0001) | 57.92 (<0.0001) | **6.84 (0.015)** | 0.060 | **<0.0001** |
| Rotarod latency | Baseline | 1.60 (0.218) | 0.04 (0.844) | 2.59 (0.121) | 0.964 | 0.104 |
| Rotarod latency | Test 1 | 1.22 (0.279) | 0.62 (0.438) | 1.40 (0.248) | 0.998 | 0.222 |
| Rotarod latency | Test 2 | 0.01 (0.936) | 5.42 (0.029) | 0.92 (0.348) | 0.790 | 0.719 |
| Rotarod latency | Test 3 | 17.16 (0.0004) | 25.41 (<0.0001) | **4.54 (0.044)** | 0.307 | **0.0003** |

**Table S5B. ABR thresholds**

| **ABR assessment** | **Frequency** | **HL main effect, F (P)** | **MPTP main effect, F (P)** | **HL × MPTP interaction, F (P)** | **Control vs HL, adjusted P** | **MPTP vs HL+MPTP, adjusted P** |
| --- | --- | --- | --- | --- | --- | --- |
| Baseline | 4 kHz | 0.27 (0.609) | 0.03 (0.863) | 0.03 (0.863) | 0.862 | 0.964 |
| Baseline | 8 kHz | 1.09 (0.308) | 0.00 (0.949) | 0.34 (0.564) | 0.937 | 0.454 |
| Baseline | 16 kHz | 0.04 (0.846) | 1.87 (0.184) | 0.34 (0.563) | 0.828 | 0.954 |
| Baseline | 22.6 kHz | 0.09 (0.761) | 0.38 (0.542) | 1.52 (0.229) | 0.491 | 0.769 |
| Baseline | 32 kHz | 3.50 (0.074) | 0.87 (0.360) | 0.22 (0.644) | 0.553 | 0.210 |
| Baseline | 45.2 kHz | 5.07 (0.034) | 1.63 (0.214) | 0.24 (0.628) | 0.400 | 0.124 |
| Post-OCI | 4 kHz | 766.95 (<0.0001) | 0.12 (0.735) | 0.12 (0.735) | <0.0001 | <0.0001 |
| Post-OCI | 8 kHz | 142.11 (<0.0001) | 0.67 (0.420) | 0.16 (0.696) | <0.0001 | <0.0001 |
| Post-OCI | 16 kHz | 1041.35 (<0.0001) | 0.22 (0.646) | 0.22 (0.646) | <0.0001 | <0.0001 |
| Post-OCI | 22.6 kHz | 470.07 (<0.0001) | 0.04 (0.834) | 0.04 (0.834) | <0.0001 | <0.0001 |
| Post-OCI | 32 kHz | 614.19 (<0.0001) | 0.14 (0.713) | 0.14 (0.713) | <0.0001 | <0.0001 |
| Post-OCI | 45.2 kHz | 846.46 (<0.0001) | 0.04 (0.849) | 0.04 (0.849) | <0.0001 | <0.0001 |

**Table S5C. TH-positive neuron counts**

| **Outcome** | **HL main effect, F (P)** | **MPTP main effect, F (P)** | **HL × MPTP interaction, F (P)** | **Control vs HL, adjusted P** | **MPTP vs HL+MPTP, adjusted P** |
| --- | --- | --- | --- | --- | --- |
| TH-positive neuron count | 132.21 (<0.0001) | 883.53 (<0.0001) | **88.71 (<0.0001)** | 0.306 | **<0.0001** |

Data were analyzed using two-way ANOVA with hearing status (HL− vs. HL+) and MPTP treatment (MPTP− vs. MPTP+) as factors. F values are reported for the main effects of HL and MPTP and for the HL × MPTP interaction. Post hoc P values were adjusted using Šidák’s multiple-comparisons test for comparisons between animals with and without HL within each MPTP condition.

**Table S6. Covariate balance before and after propensity score matching in NHANES and CHARLS.**

**Table S6A. NHANES**

| **Covariate** | **Level corresponding to reported SMD** | **Pre-matching absolute SMD** | **Post-matching absolute SMD** |
| --- | --- | --- | --- |
| Age | Continuous | 0.265 | 0.073 |
| BMI | Continuous | 0.019 | 0.099 |
| Sex | Female | 0.068 | 0.029 |
| Race | Other race | 0.237 | 0.042 |
| Marital status | Widowed/separated/divorced | 0.141 | 0.088 |
| Education | High school | 0.117 | 0.034 |
| Smoking status | Former smoker | 0.243 | 0.000 |
| Diabetes | Yes | 0.194 | 0.031 |
| Coronary heart disease | No | 0.199 | 0.074 |
| Hypertension | Yes | 0.080 | 0.093 |
| Stroke | Yes | 0.296 | 0.034 |
| Hyperlipidemia | Yes | 0.198 | 0.095 |
| Cancer | Yes | 0.047 | 0.033 |

**Table S6B. CHARLS**

| **Covariate** | **Level corresponding to reported SMD** | **Pre-matching absolute SMD** | **Post-matching absolute SMD** |
| --- | --- | --- | --- |
| Age | Continuous | 0.778 | 0.043 |
| Sex | Female | 0.026 | 0.024 |
| Marital status | Widowed/separated/divorced | 0.242 | 0.041 |
| Education | High school | 0.130 | 0.000 |
| Smoking status | Never smoker | 0.304 | 0.048 |
| Diabetes | No | 0.369 | 0.012 |
| Coronary heart disease | No | 0.763 | 0.012 |
| Hypertension | No | 0.466 | 0.000 |
| Stroke | No | 0.657 | 0.000 |
| Lipid disorder | No | 0.486 | 0.025 |
| Cancer | No | 0.106 | 0.000 |

SMDs are presented as absolute values. For continuous variables, SMDs were calculated as the difference in group means divided by the pooled standard deviation. For categorical variables, SMDs were calculated from differences in category proportions. For categorical variables with more than two levels, the maximum absolute SMD across category levels is reported, and the corresponding category is shown in the second column. An absolute SMD <0.10 was considered indicative of acceptable covariate balance. PSM was performed at a 1:1 ratio without replacement using a caliper of 0.20 standard deviations of the logit-transformed propensity score. Within the caliper, Mahalanobis distance based on the specified baseline covariates was used to select matched controls. SMD, standardized mean difference.

**Table S7. Allocation of animals to terminal RNA-sequencing and histological analyses and their final behavioral performance.**

| **Group** | **Terminal analysis** | **Open-field distance at Test 3 (cm)** | **Rotarod latency at Test 3 (s)** |
| --- | --- | --- | --- |
| Control | RNA-seq | 2108.66 | 289.19 |
| Control | Histology | 2506.45 | 300.00 |
| Control | Histology | 2101.59 | 252.91 |
| Control | RNA-seq | 2376.82 | 300.00 |
| Control | RNA-seq | 2295.42 | 275.16 |
| Control | Histology | 2098.63 | 300.00 |
| Control | Histology | 2136.64 | 283.96 |
| HL | RNA-seq | 1953.39 | 258.63 |
| HL | Histology | 2033.46 | 269.38 |
| HL | RNA-seq | 1914.62 | 269.59 |
| HL | Histology | 1901.09 | 280.32 |
| HL | Histology | 1863.55 | 274.19 |
| HL | Histology | 1883.98 | 258.48 |
| HL | RNA-seq | 1831.30 | 278.45 |
| MPTP | Histology | 1932.39 | 214.30 |
| MPTP | Histology | 2144.63 | 279.65 |
| MPTP | RNA-seq | 2192.34 | 297.27 |
| MPTP | Histology | 1599.28 | 261.82 |
| MPTP | RNA-seq | 1364.17 | 259.65 |
| MPTP | Histology | 1368.31 | 266.72 |
| MPTP | RNA-seq | 1574.88 | 259.52 |
| HL+MPTP | Histology | 406.88 | 203.43 |
| HL+MPTP | RNA-seq | 781.37 | 262.98 |
| HL+MPTP | Histology | 1115.45 | 223.72 |
| HL+MPTP | Histology | 737.94 | 218.45 |
| HL+MPTP | Histology | 1304.77 | 174.27 |
| HL+MPTP | RNA-seq | 1310.08 | 210.20 |
| HL+MPTP | RNA-seq | 667.04 | 196.05 |

All seven mice in each experimental group completed behavioral testing. Following the final behavioral assessment, three mice per group were allocated to striatal RNA sequencing and the remaining four mice per group to histological analysis. The table presents the final open-field and rotarod measurements of the corresponding animals. RNA-seq biological replicates consisted of tissue from individual mice without pooling.

**Figure S1. Baseline auditory brainstem response thresholds in the four experimental groups before intervention.**
